# Pushing ML Predictions Into DBMSs

**DOI:** 10.1109/TKDE.2023.3269592

**Published:** 2023-04-24

**Authors:** Matteo Paganelli, Paolo Sottovia, Kwanghyun Park, Matteo Interlandi, Francesco Guerra

**Affiliations:** University of Modena and Reggio Emilia 9306 41121 Modena Italy; Huawei Research Munich 474238 80992 München Germany; Yonsei University 26721 Seoul 03722 South Korea; Microsoft Research 214606 Redmond WA 98052 USA

**Keywords:** MLOPs, machine learning, SQL

## Abstract

In the past decade, many approaches have been suggested to execute ML workloads on a DBMS. However, most of them have looked at in-DBMS ML from a training perspective, whereas ML inference has been largely overlooked. We think that this is an important gap to fill for two main reasons: (1) in the near future, every application will be infused with some sort of ML capability; (2) behind every web page, application, and enterprise there is a DBMS, whereby in-DBMS inference is an appealing solution both for efficiency (e.g., less data movement), performance (e.g., cross-optimizations between relational operators and ML) and governance. In this article, we study whether DBMSs are a good fit for prediction serving. We introduce a technique for translating trained ML pipelines containing both featurizers (e.g., one-hot encoding) and models (e.g., linear and tree-based models) into SQL queries, and we compare in-DBMS performance against popular ML frameworks such as Sklearn and ml.net. Our experiments show that, when pushed inside a DBMS, trained ML pipelines can have performance comparable to ML frameworks in several scenarios, while they perform quite poorly on text featurization and over (even simple) neural networks.

## I. Introduction

In The last few years, the interest in Machine Learning (ML) both in academia (approximately 100 new ML-related papers are published on Arxiv every day [1]) and in the industry [2], [3], [4], [5], [6], [7], [8], [9], [10], [11], [12], [13], [14], [15] has exploded. The expectation is that, in the near future, every application will incorporate trained ML models for all those functions that are impossible to write for software developers [2]. To fulfill this vision, ML has to transition from art and science into a mature engineering discipline [16] centered around data [17]. Unfortunately, it is remarkably easy to accumulate massive maintenance costs (referred to as *technical debt*) at the system level when ML is used [18].

For the last 4+ decades, Database Management Systems (DBMSs) have proven to be the workhorse of many enterprises. Governance, security, audibility, access control, provenance, and performance are some of the common features found in “Enterprise-grade” software such as DBMSs. One natural question then arises: to which extent can DBMSs be used to lower the technical debt of ML deployments and achieve *Enterprise-grade ML* [16]? Many works [19], [20], [21], [22], [23], [24] have indeed already explored this problem, although mostly from an ML training perspective, or for a few model classes. Conversely, in-DBMS prediction serving of *end-to-end ML pipelines* (i.e., pipelines composed of featurizers and ML models) remains largely an open question. This is somehow surprising, in fact:

1) in practice, ML models are seldomly deployed alone, whereas data featurizers are often required to transform data into the format that is understandable by ML models (e.g., in [25] we found that pipelines can have up to hundreds of operators);
2) models are often trained once and served many times (e.g., rendering of web pages based on users’ profiles, batch prediction of asset prices based on historical data), and this pattern appears quite amenable for in-DBMS execution;
3) applications where prediction serving will likely be used (e.g., websites, smart BI dashboards) are often backed by a DBMS;
4) the top used operators in practical data science over tabular data are not compute-heavy neural networks, but rather memory-intensive operations (such as one-hot encoding or tree ensemble methods [25], [26]) which should benefit from in-DBMS execution;
5) when data already resides in a database, execution of in-DBMS predictions is a natural choice, whereas a different solution will require pulling the data out of the database. This not only is a path not always practicable, for instance, if for security reasons data cannot be moved outside the database, but it also causes performance costs, while making it difficult to enforce the “Enterprise-grade” features without resorting to bespoken solutions (and likely increasing the technical debt).

Our observation is further corroborated by the fact that commercial databases are starting to surface functionalities for expressing model predictions directly from SQL statements [27], [28], [29], [30]. Pushing the execution of predictions directly into the DBMS by translating ML pipelines end-to-end into SQL is therefore the natural next step.

To study whether trained ML pipelines can be pushed into DBMSs, and predictions served directly in SQL, we have collected 10 representative pipelines, spanning (1) different ML tasks (binary, multiclass classification, and regression); (2) a diverse set of models (linear, tree ensembles) and featurizers (one-hot encoder, normalizer, etc.); and (3) a heterogeneous set of datasets (from large scale with 10 s of millions of records to small ones with only few 100 s instances). We experimentally evaluate the performance of Sklearn and ml.net pipelines against their SQL implementations executed over MySQL and SQL Server; we evaluate the performance of using both different input / output modalities (flat CSV file or database), prediction settings (batch or online), and optimization and implementation strategies (e.g., w/ and w/o indexes, columnar store, operator fusion). SQL implementations are generated by ${\sf MASQ}$MASQ (*Machine learning AS Query*): a library whereby trained ML pipelines are translated into standard SQL (without UDFs or vendor-specific syntax) and are therefore executable on any DBMSs.

Our experiments show that DBMSs performance can be comparable to Sklearn and ml.net when data resides in the database. Conversely, when ML pipelines contain textual featurizers or compute heavy models (e.g., neural networks) databases perform quite poorly. Summarizing, the contributions of the paper are:

• We introduce ${\sf MASQ}$MASQ^fn1^^1^https://github.com/softlab-unimore/MASQ: a library able to translate trained ML pipelines into SQL;
• We empirically evaluate the performance of queries implementing trained ML pipelines on a diverse workload, and compare in-DBMS predictions against two ML frameworks;
• We provide a set of lessons learned (e.g., how to circumvent the limit on the number of database columns), and additional insight related to running ML predictions natively on DBMSs.

To our knowledge, we are the first to evaluate the performance of ML pipelines run end-to-end in plain SQL and to show that SQL execution can be achieved even for high-dimensional ML models and featurizers going beyond DBMSs limits.

The paper is organized as follows: Section II sets the background. Related works are listed in Section III. Section IV describes ${\sf MASQ}$MASQ implementation. The experiments are in Section V. The paper ends with lessons learned and conclusions in Section VI.

## II. Background: ML Workflow

**Fig. 1.**
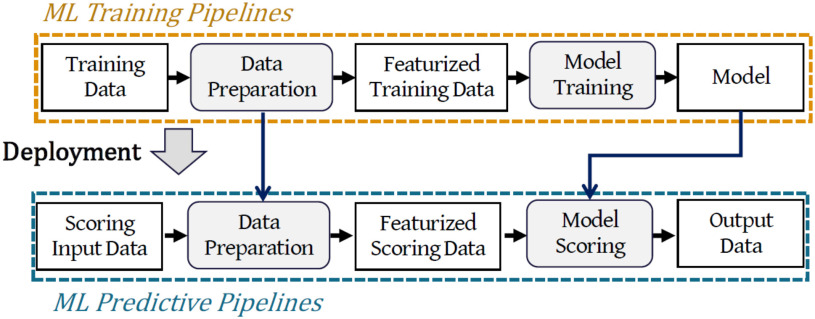
A typical ML workflow. Rectangles are used to identify data artifacts (e.g., input data, or trained models); ellipses determine computations (e.g., data preparation and serving).

Fig. 1 depicts a typical ML workflow. Starting with some *input data*, a *data preparation* step is used for sanity checks, data validation, data cleaning, feature generation, and selection. Data preparation is commonly performed through a set of *data featurizers*. The *featurized data*, the output of the data preparation step, is then passed to the *training* step, where a *learning* algorithm is used to fit an ML model through an iterative process. Once the model is trained, it can be represented as a *prediction function* transforming input features into a prediction score (e.g., 1 or 0 for binary classification). Finally, the trained ML model along with the data preparation operators constitute the *ML predictive pipeline* which is then *deployed* for *serving* prediction queries [31]. Wrapping data preparation and trained models into a unique artifact is common practice in ML systems [2]. At serving time, the new input data is pre-processed and featurized (using the same operators) and fed into the prediction function of the trained ML model for rendering the final score.^fn2^^2^This is an oversimplification of actual ML workflows, and it does not cover, for example, hyper-parameter tuning and model selection. It is however a fair summary of common use cases. In this work, we deal only with “pure” pipelines, i.e., pipelines composed only of Sklearn or ml.net operators, and without arbitrary code.

*The focus of this article is to study whether the prediction serving process can be pushed down and directly executed on DBMSs.* The training process is kept as in the typical ML workflow and is not the focus of this article. Rather, once a model is trained, we use ${\sf MASQ}$MASQ to generate SQL queries that perform the same data preparation and prediction logic as the original predictive pipeline. We purposely target standard SQL such that we can (1) target different DBMSs; and (2) allow the optimizer to properly generate efficient end-to-end plans. Finally, our focus is on models learned over relational data. Therefore, we will only consider pipelines composed of “traditional” ML operators. (i.e., no deep neural networks). Traditional methods are the state-of-the-art over structured data [32], and it is still the more widely-used type of ML [25], [26]. Nevertheless, we did test the performance of a shallow neural network in Section V-H2.

## III. Related Work

The integration of ML into RDMBSs has a long history. In the early 2000 s, SQL Server shipped with data mining operators for classification and clustering [33]. Later in the 2010 s, MADlib [19] and following works (e.g., [20], [34], [35], [36]) propose to use User-Defined Aggregates (UDAs) and User-Defined Functions (UDFs) as the Trojan Horse to overcome the limitations of DBMS regarding iterative computation and linear algebra routines. Apache Spark's MLlib [37], SystemML [14], [15], Apache Mahout Samsara [38] and others [39], [40] could be seen as a continuation of this trend. Beyond UDAs/UDFs, other approaches have tried to add ML to DBMSs by extending database runtimes with linear algebra operations (e.g., [41], [42]). While extending consumer database runtimes for properly supporting ML will bring the best performance, this is a herculean task because it requires the modification of decades-old systems. Conversely, the UDA/UDF approach is more generic, but it introduces non-trivial overheads [43], while limiting the set of possible cross-optimization between ML and relational algebra [44], [45]. Finally, factorized approaches (e.g., [46]) rewrite ML models in a database-friendly way. While these approaches work well over linear-algebra-based models, it is not clear whether they can also support effectively tree-ensemble models.

All the above-mentioned works mostly focus on (1) the training aspect of ML, and on (2) optimizing specific workloads relying heavily on linear algebra. Conversely, our focus is far less ambitious but arguably practical: we want to *understand whether off-the-shelf DBMSs are a good fit for serving ML predictive pipelines*. Our observation is that, in practice, predictive pipelines are not deployed into DBMSs, but rather into external containers [47] or directly into the application [48], *even when input data resides in a relational format in a database*. Furthermore, predictive pipelines are composed of a variety of prediction functions and data featurizers (e.g., tree methods and one-hot encoding), where inefficient linear algebra operations are not necessarily the bottleneck. Tidypredict [49] is probably the closest work to ${\sf MASQ}$MASQ, although it works only in R, and for a small set of models (linear regression, generalized linear model, random forest, and decision tree). Amazon Redishift ML [29] and Azure Synapse Analytics allow SQL predictions, but this is achieved by a wrapper around external libraries. Google's BigQuery supports inference (and training) directly in SQL, but only for generalized linear models [50]. Interestingly, the original version of MADlib [51] did follow the same “pure” SQL approach of ${\sf MASQ}$MASQ. However, they found that *“Unfortunately, the portable core of* vanilla*SQL is often not quite enough to express the kinds of algorithms needed for advanced analytics.”* [19]. Nonetheless, this argument refers to training, while inference algorithms are in general simpler. More recently, [52] also proposed to translate ML training into SQL, and with really good performance. Raven [44] co-optimizes predictive pipelines and SQL queries. Among the optimizations, Raven can generate SQL queries from ML operators. We see our study in this article as complementary to approaches such as Hummingbird [53] since we specifically target predictive pipelines when data resides on a database and no hardware accelerator is available. Even when hardware accelerators are available, using Hummingbird requires (1) pulling the data from the database; (2) transforming the data into columnar format; (3) transferring the data into GPU memory (and back). Executing predictive pipelines directly into SQL can therefore still be widely beneficial because all the above steps can be avoided. The cross-point on when one technique is better than the other is investigated elsewhere [54].

In this article, we will focus on traditional ML, and compare DBMS execution against Sklearn [55] and ml.net [2]. Other alternative libraries include H2O [56], Weka [57], and Spark's MLlib [37] (for scale-out training). A demonstration of ${\sf MASQ}$MASQ functionalities was presented at SIGMOD 2021 [58].

## The ${\sf MASQ}$MASQ Library

IV.

**Fig. 2.**
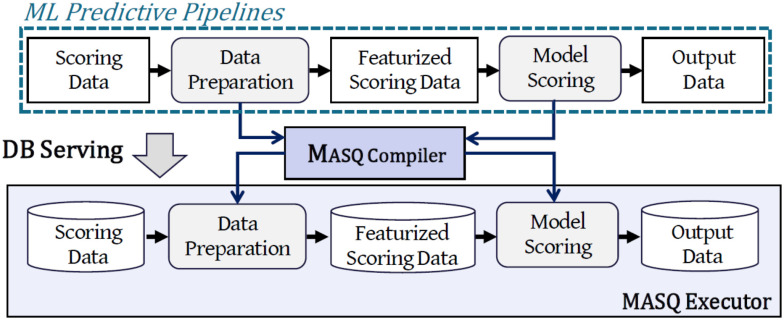
${\sf MASQ}$MASQ applied to an ML predictive pipeline.

As a first step, we describe how trained pipelines can be programmatically translated into SQL. The ${\sf MASQ}$MASQ library consists of two main components (Fig. 2). The *Compiler* (Section IV-A) is responsible for the transformation of the predictive pipelines into SQL queries; the *Executor* (Section IV-B) instead connects and runs the queries on the DBMS holding the data.

### A. The Compiler

The *Compiler* job can be divided into three phases: during *parsing* (Section IV-A1) the fitted parameters are extracted from the trained featurizers and models; parsed pipelines are then *analyzed* (Section IV-A2); finally, a *conversion* phase (Section IV-A3) generates the SQL implementations.

#### 1) Parsing

Predictive pipelines are actually Direct Acyclic Graphs (DAGs) of *operators*, where each operator can be a data featurizer or a model. In the parsing phase, input predictive pipelines are parsed one operator at a time, and each operator is *wrapped* by a *container* object maintaining input/output relationships, as well as an *operator signature* and an *extractor function* used for extracting the fitted parameters. Operator signatures are initialized with the object types (e.g., the result of the type function applied over a Python operator object) and used for picking the correct extractor (and conversion) function for the given operator instance. ${\sf MASQ}$MASQ compiler is extensible: extractor functions are registered at startup time into a hash table mapping operator signatures into the related extractor function. In its current implementation, ${\sf MASQ}$MASQ provides wrappers for the Sklearn and ml.net libraries, and extractors for linear and tree models, as well as a handful of featurizers (standard scaler, one-hot encoder, and label encoder). At the end of the parsing phase, the input pipeline is “logically” represented in ${\sf MASQ}$MASQ as a DAG of containers storing all the information required for the successive analysis and conversion phase.

**Example 1 (Parsing a Sklearn Pipeline).**

Let us suppose that a user provides a Sklearn pipeline composed of a *scaler* [59] followed by a *linear regression* model. Furthermore, let us suppose that the pipeline is applied over the numeric columns of the ${\sf TaxiTable}$TaxiTable dataset represented in Table I

**TABLE I:** The ${\sf TaxiTable}$TaxiTable Used in the Examples. Abbreviations: Pc = Passenger Count; Tts = Trip Time; Td = TripDistance; Pt = Payment Type; Vi = Vendor Id. The Label is the Fare Amount

	${\sf Pc}$Pc	${\sf Tts}$Tts	${\sf Td}$Td	${\sf Pt}$Pt	${\sf Vi}$Vi		${\sf Label}$Label
$t_{1}$t1	1	1271	3.8	crd	cmt		17.5
$t_{2}$t2	1	720	2.34	crd	vts		10.5
$t_{3}$t3	1	0	11.06	csh	vts		120
$t_{4}$t4	1	3	0	noc	cmt		52
$t_{5}$t5	5	1560	19.97	unk	vts		52

. Fig. 3

**Fig. 3.**
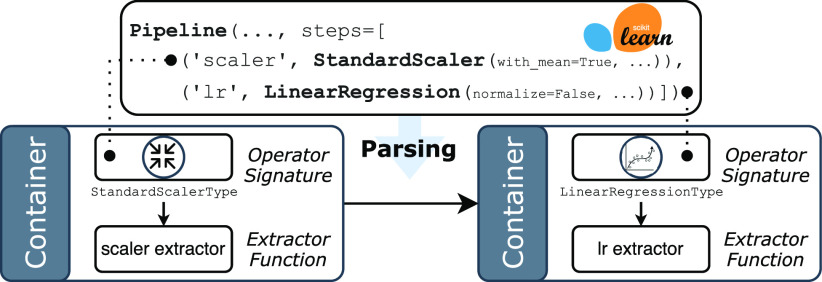
Parsing of the pipeline of Example 1. The pipeline (top) is parsed on a container DAG (bottom). Each container stores a reference to the operator, its signature and extractor.

depicts the trained pipeline object with an excerpt of its parameters (top) and the result of parsing (bottom). During parsing ${\sf MASQ}$MASQ (1) generates a container wrapping each operator, and containing the extractor function; and (2) wires the containers into a DAG following the input/output dependencies in the pipeline (in this specific example, the container DAG is a simple sequence).

#### 2) Analysis

In this phase, the DAG of containers generated in the parsing phase is traversed in topological order. During the traversal pass, for each operator ${\sf MASQ}$MASQ extracts the operator's parameters by calling the referenced extractor function stored in the container. Extracted parameters are stored within the container. ${\sf MASQ}$MASQ supports different to-SQL *converters* based on the operator characteristics. By default, ${\sf MASQ}$MASQ uses a mix of select and case statements for converting ML operators into SQL (Section IV-A3), but sometimes the number of features or structure of the operators is restricted by DBMSs’ limits. In the latter case, in the first traversal pass, ${\sf MASQ}$MASQ rewrites the queries in order to bypass the database limitations. We will show in Section IV-A4 a couple of techniques used by ${\sf MASQ}$MASQ for this task.

**Example 2 (Analysis of the Sklearn Pipeline).**

During analysis, the extractor functions of the parsed pipeline of Example 1 are triggered. Specifically, the parameters extracted from the scaler and linear model are shown in Tables II(a) and II(b), respectively. In the StandardScaler case, the extractor pulls the *mean* and the *standard deviation* values for each column by calling mean_ and scale_ from the operator object, respectively. The extractor for the LinearRegression retrieves the weights and the bias by calling respectively operator.coef_ and .intercept_.

**TABLE II:** Parameters Extracted From the Pipeline of Example 1

	${\sf Pc}$Pc	${\sf Tts}$Tts	${\sf Td}$Td
$mean$mean	1.8	710.8	7.434
$std$std	1.6	638.9	7.28
(a) Scaling			

${\sf Findex}$Findex	${\sf Weight}$Weight		
1	-20.33		
2	-31.36		
3	48.72		
bias	50.4		
(b) Regression

#### 3) Conversion

During this last phase, the DAG of containers is again traversed in topological order and a conversion-to-SQL function is triggered based on each operator signature. Each conversion function receives as input the parameters (extracted during analysis, and stored directly into the container) and generates a string containing the SQL implementation. The SQL implementations of all operators are then merged into a unique query following the input/output dependencies expressed in the container DAG.

As for the extractors, ${\sf MASQ}$MASQ stores a map of the operator signatures/conversion functions. ${\sf MASQ}$MASQ currently implements converters for the following operators (where each of them has a default and triplet-format version): standard scaler, one-hot encoder, label encoder, gradient boosting classifier/regressor (w/ and w/o tweedie loss), random forest, decision tree, linear regression with some variants (i.e., Poisson and SDCA), logistic regression classifier, PCA, and linear SVM classifier. In the default case, the above operators can be implemented using the following simple strategies.

##### a) Conversions via select statements

The conversion into SQL is straightforward when the ML prediction function consists only of algebraic operations between the extracted parameters and the input features. Examples of methods implemented via select statements are normalizers/scalers and linear models (by unrolling the linear algebra operations into the select clause).

**Example 3 (Pipeline conversion).**

**Fig. 4.**
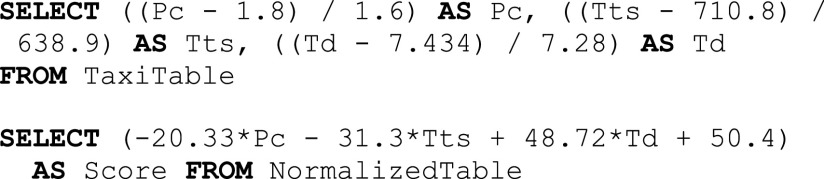
Scaling and linear model in SQL.

The conversion of the pipeline of Examples 1 and 2 leads to the queries in Fig. 4, where the two select clauses implement the scaler and the regressor, respectively. For the former case, scaling is implemented by subtracting and subsequently diving each column by the pre-defined values generated during training. For the latter case, the weights and bias of the linear regression model are multiplied with the corresponding column, and the bias term added afterward. Note that the queries, at conversion time, will be merged into a unique query.

##### b) Conversions via case statements

SQL case statements can be used to implement rule-based learners such as decision trees, or data featurizers such as one-hot encoding (OHE). In the former case, each rule from the model is translated into a SQL case statement; rules are then nested, according to the model, by nesting the correspondent case statements. For the latter, we use case statements to encode input categorical values into a sequence of columns, one for each distinct value. For each input, only the column of that particular categorical value will store 1, all the other columns will be 0.

**Example 4 (OHE).**

**Fig. 5.**
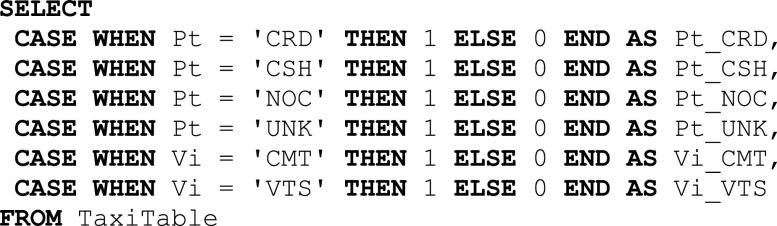
One-hot encoding in SQL.

We want to apply a one-hot encoder to the columns ${\sf Pt}$Pt and ${\sf Vi}$Vi of the data represented in Table I. The result of this transformation is a new set of columns, one for each unique categorical value of the ${\sf Pt}$Pt and ${\sf Vi}$Vi columns. As we can see in the query of Fig. 5, each column name is generated by concatenating the original categorical input name with each distinct value. Each column will store 1 only if the value is of the proper category.^fn3^^3^Note that this example is only for providing a high-level description of how OHE could be implemented in SQL. In ${\sf MASQ}$MASQ we use a “sparse” version of the above example where only non-zero values are materialized.

##### c) Combining select and case statements

Some model requires the combination of select and case statements. This is, for example, the case for tree ensemble models. Tree ensemble methods construct a sequence of decision trees and adopt different strategies to select the output class (e.g., the mode class in classification tasks, and the means of the resulting values in regression tasks). In the SQL implementation for this kind of method, we nest the case-based queries of the decision trees in a query that collects the results and computes the final output via a select clause.

#### 4) Escaping DBMSs’ Limits

DBMSs are not designed for ML, and it is fairly easy to reach database limits with ML pipelines of reasonable complexity. During the analysis phase, ${\sf MASQ}$MASQ detects when a certain limit is reached, and it automatically selects, at conversion time, the proper operator implementation. Next, we list a couple of problems, and related solutions, we encountered while implementing ${\sf MASQ}$MASQ.

*Limit to the number of columns.* SQL Server-wide (sparse) tables support 30 k columns; 1024 in regular tables [60]. MySQL supports a maximum of 4096 columns per table [61]. Conversely, ML datasets and pipelines can easily reach several millions of features. Therefore, high dimensional data needs to be stored using a different format.

${\sf MASQ}$MASQ *solution.* To overcome the above problem, we use a *triplet-based representation* where each record is stored in the form (identifier, attribute_name, attribute_value). In the analysis phase, ${\sf MASQ}$MASQ injects a *triplet-representation operator* (TRO) into the plan if the number of columns is too large. This operator is used to inform the compiler to transform the data from the default into a triplet format during the conversion phase and to successively use the related triplet-based conversion function for each subsequent operator. As an example, next, we show the compilation process for a pipeline that contains an OHE operator generating a large number of features.

**Example 5 (Pipeline with TRO and OHE).**

**Fig. 6.**
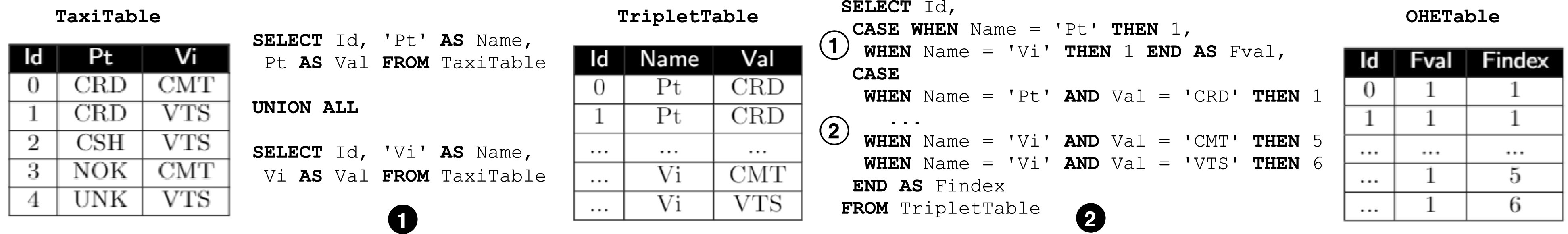
SQL workflow for the one-hot encoding sparse implementation.

Let us assume we want again to transform the columns ${\sf Pt}$Pt and ${\sf Vi}$Vi of Table I using OHE. This time, however, we assume that the total number of distinct values for these categorical columns is greater than the maximum number of columns supported by the database.^fn4^^4^This check is, for example, implemented for Sklearn as a condition on the total number of elements of the parameter extracted from operator.categories_. In this case, the compiler will inject a TRO operator before OHE. The following converter is then instructed to use the triplet-based conversion function for OHE, which uses a sparse implementation. Specifically, the converter in this case generates pairs in the form (1, index_value) instead of materializing the full dense vector as we did in Example 4. In Fig. 6

we provide the SQL workflow implementing the pipeline. The SQL statement on the left-hand side of the Figure (➊) implements the TRO operator. This creates a ${\sf TripletTable}$TripletTable where the first column is the identifier of the rows in the dataset, while the second and third columns store the attribute name and its values, respectively. In the SQL query on the right-hand side (➋), the first case statement (①) is used to select the attribute(s) to encode and sets 1 as the value for those attributes.^fn5^^5^Note that even if ${\sf Fval}$Fval contains all 1 s and therefore could be removed, we keep them to maintain a uniform interface across the operators defining the predictive pipelines. The second case statement (②) provides the index of non-zero values. Note that indexes are sequential, even across categorical columns (the index for the ${\sf Vi}$Vi column starts at 5 instead of 1). This is because we implicitly *concatenate* one-hot encoded columns into a unique feature vector.

*Limits on* select *and* case *clauses.* High-dimensional datasets introduce problems not only regarding the data representation but also regarding how we implement operators in SQL. In fact, limits exist on the number of columns allowed in select statements (e.g., 4096 for SQL Server), or the total number of conditions in case clauses (few thousand for SQL Server [62]).

${\sf MASQ}$MASQ *solution.* These two issues are addressed by injecting TROs, and partitioning large select and case statements.^fn6^^6^Currently, the partitioning strategy takes track of how many elements each statement contains, and it creates a new query once the number of statements surpasses the database maximum. We show how this strategy works through two pipelines made of an OHE plus a linear regression (Example 6) and a tree ensemble (Example 7).

**Fig. 7.**
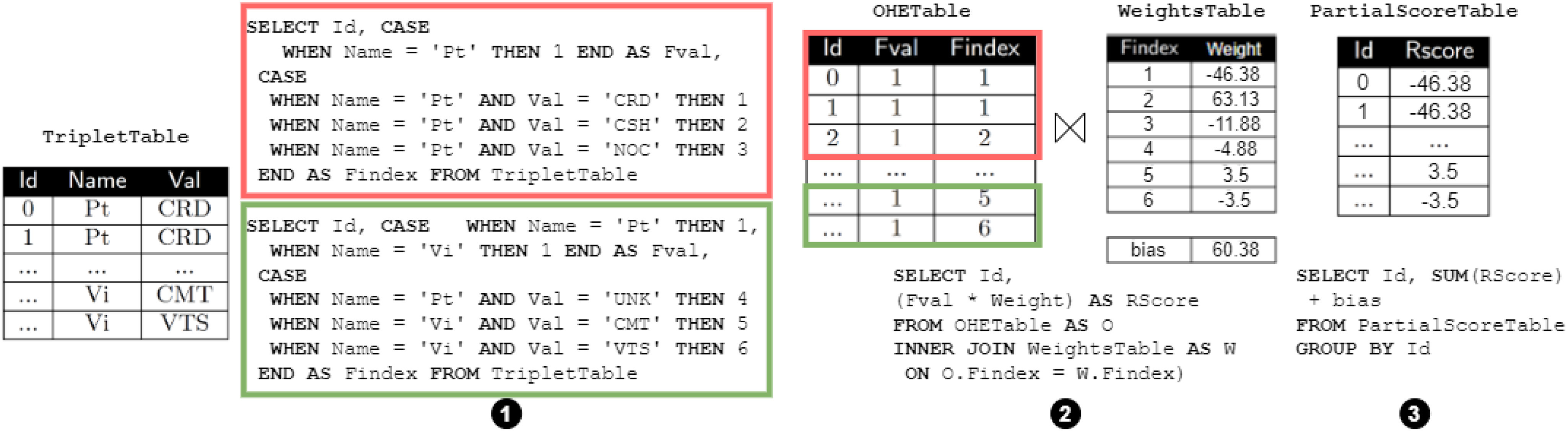
Pipeline with OHE followed by a linear regression executed in ${\sf MASQ}$MASQ with TRO and partitioning.

**Example 6 (OHE and linear regression).**

Fig. 7 depicts how ${\sf MASQ}$MASQ translates this pipeline. Due to space constraints, we directly start from the ${\sf TripletTable}$TripletTable of Example 5, because, as in the previous example, we assume that the OHE generates a large number of features. Additionally, we assume that also the number of case statements in the OHE is too large, and therefore the query for the encoding needs to be partitioned (➊). Each partition is executed independently and generates a distinct ${\sf OHETable}$OHETable. The ${\sf OHETable}$OHETables are then joined (➋) with the ${\sf WeightsTable}$WeightsTable containing the linear regression's parameters (e.g., Table II(b)). Over the output of the join, we then multiply each feature value with the respective regression weight, and generate the partial sums which will then be aggregated by a final query (➌). Note that, differently than the unrolled version of Section IV-A3a, by using the triplet representation we can also avoid the limit of columns in the select statements.

**Example 7 (OHE and tree ensemble model).**

**Fig. 8.**
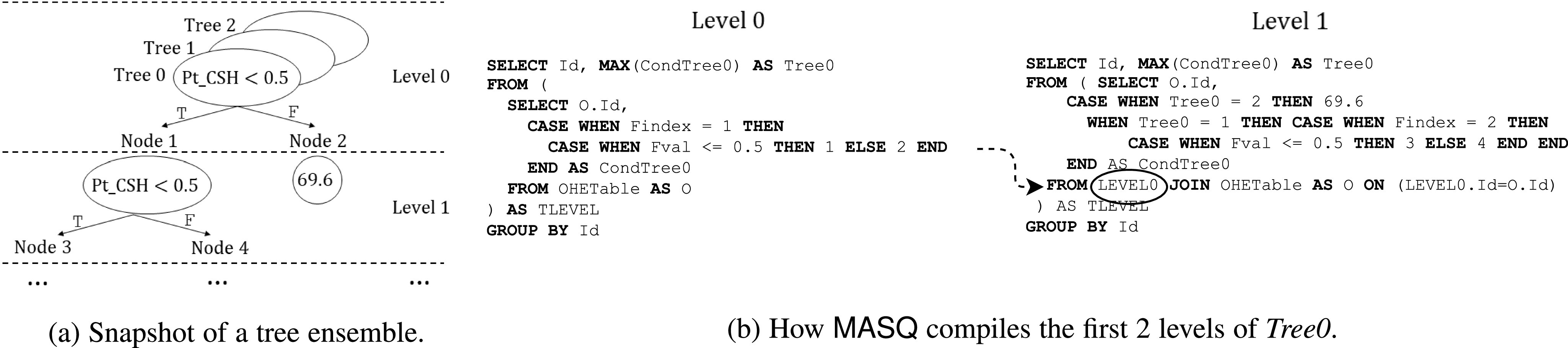
How tree ensembles over triplet are translated in ${\sf MASQ}$MASQ.

The implementation of tree ensemble models after OHE basically follows the same workflow as Example 6, with two important differences. First, while ${\sf WeightsTable}$WeightsTable can be partitioned following the ${\sf OHETable}$OHETable partitioning, for tree ensembles each tree could potentially touch all input features. To solve this, we partition tree ensembles into *batches* (up to the number allowed by DBMS constraints), and run each batch over the union of the ${\sf OHETable}$OHETables. Second, case statements cannot be directly used to implement trees on data in triplet-based representation. This is because each original (not triplet) row is split into several triplet rows, and case statements, to work, should now be able to select multiple rows simultaneously. To overcome this limitation, ${\sf MASQ}$MASQ implements a technique whereby all the trees in the batch are traversed together, level by level, in a breadth-first search manner. For each level, we select the triplets that match the conditions on the trees, and we use the condition to select the next case statement in the next level. In Fig. 8(a)

we depict a tree ensemble model with 3 trees and we detail specifically the first 2 levels of *Tree0* where the decision nodes use OHE features ${\sf Pt_CRD}$PtCRD and ${\sf Pt_CSH}$PtCSH. Fig. 8(b) contains the queries for the first 2 levels of *Tree0*. The query for *Level 0* (i.e., the root) of *Tree0* contains two nested case statements: one for selecting the proper feature (i.e., feature ${\sf Pt_CRD}$PtCRD has ${\sf Findex}$Findex = 1, ${\sf Pt_CSH}$PtCSH has ${\sf Findex}$Findex = 2), and one for evaluating the condition of the feature. The result of the condition contains the index of the node which will then be used in the successive level. The final group by and max operations are used to return a unique not null record. In *Level 1* query we use the results of *Level 0* and have three nested case statements: in the outermost statement we have one condition for each node, while for each node we have, again, two ${\sf case}$case statements, one for selecting the proper feature, and one for evaluating the condition. The other levels follow a similar approach. With this technique, we can evaluate, for each level, batches of trees concurrently. This algorithm is equivalent to Tree Traversal strategy in [53]. The SQL query of Fig. 8(b), for each level, will then actually contain different case statements for each tree. We add padding logic to deal with trees with different numbers of levels.

### B. The Executor

The *Executor* provides the functionalities necessary for the execution of the SQL queries generated by the *Compiler* in a relational database. The *Executor* also makes use of a set of connectors (currently we support MySQL and SQL Server via Python and C# connectors) for extracting the data from the database and running the original pipeline externally as validation. Finally, a small *driver* program manages the executions of the pipelines (either as a SQL query or externally to the database). We refer readers to our demo paper [58] for a visualization of the execution flow in ${\sf MASQ}$MASQ.

## V. Experimental Evaluation

The main question we want to answer in this experimental evaluation is: *are databases a good fit for serving ML predictive pipelines?* To answer this question, we (1) select 10 representative ML pipelines; (2) implement them on two ML frameworks, namely Sklearn and ml.net; and (3) compare their execution against ${\sf MASQ}$MASQ-generated queries run on 2 DBMSs: MySQL and SQL Server. We test both the final accuracy (with the expectation of matching the same accuracy of the ML frameworks), the throughput, and the latency performance over single record predictions. Finally, we (4) further explore how SQL pipelines perform with optimizations such as indexing and operator fusion; and (5) report some negative results on text featurization and neural network models. The experiments are organized as summarized in Table III.

**TABLE III:** Description of the Experiments

Experiment	Goal
Accuracy (Section V-A)	Evaluation of the conformity of the final predicted output
Throughput (Section V-B)	Runtime performance comparison among the frameworks
Scalability (Section V-C)	Evaluation of the time performance as the data batch size changes
Latency (Section V-D)	Performance when executing prediction over a single record
Performance Breakdown (Section V-E)	Evaluation of where the time is spent (1) per-operator; (2) within each operator on load, computing, and write operations
Optimizations (Section V-F)	Evaluation of the impacts of optimizations such as adding indexes or operator fusion
Operators Implementation (Section V-G)	Study of possible variants for the SQL translations
Special Cases (Section V-H)	Study of additional pipelines for textual data and Neural Network Models

*Datasets.* For the main experimental evaluation, we employed 7 datasets (see column *Dataset Size* in Table IV for details). On these datasets, we run a wide range of tasks: from binary and multi-class classification to regression. ${\sf Iris}$Iris is the smallest one with 150 records, each described by 4 numeric columns. ${\sf Criteo}$Criteo is the dataset with the largest number of features (39 columns). At prediction time the input columns are transformed with OHE into around 2.5 million features. ${\sf Flight Delay}$FlightDelay is the biggest dataset: it contains more than 21 million records and 26 initial columns which, during execution, they get expanded into approximately 700 features.

**TABLE IV:** Pipelines and Datasets Used in the Experiments. In Brackets, the Effectiveness in Terms of Accuracy for Classification, $R^{2}$R2 for Regression Pipelines. Abbreviations: FTC = FastTreeClassifier; FTR = FastTreeRegression; FTT = FastTreeTweedie; GBDTR= GradientBoostingRegression; GBDTC= GradientBoostingClassifier; LBFGSR = LBFGSPoissonRegression; LR = LogisticRegression; SDCAR = SDCARegression; SDCAME = SDCAMaximumEntropy SGDR = SGDRegression; DC= DropColumns; DF= DropFeatures; LE = LabelEncoder; MVK= MapValueToKey; NMV = NormalizeMeanVariance; SS = StandardScaler; Sel St = Select Statement; Case St = Case Statement; PC = Partitioned Case Statement; PCS = Partitioned Case, Select Statement

Pipeline	Dataset Size	Featurizer	Model	Pipeline	Dataset Size	Featurizer	Model	Framework
**P1** ${\sf Iris}$Iris**(acc. 0.98)**	150 rows 4 cat. cols	MVK	SDCAME	**P2** ${\sf Heart Disease}$HeartDisease**(acc. 0.95)**	303 rows 13 num. cols	N/D	FTC	ml.net
		LE	LR				GBDTC	Sklearn
		Sel St	Sel St				Case St, Sel St	${\sf MASQ}$ MASQ
**P3** ${\sf Bike Sharing}$BikeSharing** ($R^{2}$R2 0.33)**	17,379 rows 12 num. cols	N/D	LBFGSR	**P4** ${\sf Bike Sharing}$BikeSharing** ($R^{2}$R2 0.27)**	17,379 rows 12 num. cols	N/D	SDCA-R	ml.net
			SGDR				SDCAR	Sklearn
			Sel St				Sel St	${\sf MASQ}$ MASQ
**P5** ${\sf Bike Sharing}$BikeSharing** ($R^{2}$R2 0.88)**	17,379 rows 12 num. cols	N/D	FTR	**P6** ${\sf Bike Sharing}$BikeSharing** ($R^{2}$R2 0.91)**	17,379 rows 12 num. cols	N/D	FTT	ml.net
			GBDTR				XGBoost	Sklearn
			Case St, Sel St				Case St, Sel St	${\sf MASQ}$ MASQ
**P7** ${\sf Taxi Fare}$TaxiFare** ($R^{2}$R2 0.57)**	200,000 rows 3 num. cols 3 cat. cols	OHE, NMV	SDCAR	**P8** ${\sf Credit Card}$CreditCard** (acc. 0.99)**	284,897 rows 30 num. cols	DC, NMV	FTC	ml.net
		OHE, SS	SDCAR			DF, SS	GBDTC	Sklearn
		Case St, Sel St	Sel St			Sel St	Case St, Sel St	${\sf MASQ}$ MASQ
**P9** ${\sf Criteo}$Criteo** (acc. 0.73)**	4,000,000 rows 13 num. cols 26 cat. cols	OHE	FTC	**P10** ${\sf Flight Delay}$FlightDelay** ($R^{2}$R2 0.99)**	21,604,865 rows 23 num. cols 3 cat. cols	OHE	FT-R	ml.net
		OHE	GBDTC			OHE	GBDTR	Sklearn
		TRO + PC	PCS			TRO + PC	PCS	${\sf MASQ}$ MASQ

*ML Pipelines.* Table IV shows the pipelines we will be using in our evaluation. 8 pipelines have been taken from ml.net samples [63]; 2 of them (${\sf Criteo}$Criteo and ${\sf Flight Delay}$FlightDelay) are pipelines commonly used to evaluate the scalability of ML frameworks [2]. For each pipeline, we (1) started with an implementation in ml.net; (2) we re-implemented it over Sklearn (note that for P3 we used XGBoost [64] as GBDT library in order to match the Tweedie loss on ml.net); and finally (3) we used ${\sf MASQ}$MASQ to generate SQL queries for both implementations. For each pipeline, Table IV contains the *Featurizer*s (when used) and the final *Model*. For each pipeline, we list the used featurizers and models by *Framework*; for ${\sf MASQ}$MASQ we mentioned which technique we used from Section IV, i.e., whether we used select statements, case statements, both select and case statements, TROs, or partitioned statements. Finally, the Table reports the effectiveness of the pipelines (in terms of accuracy for classification models and $R^{2}$R2 score for regression models) as computed with the ml.net framework.

*Setup.* The experiments are executed on an Azure Standard D32 v3 machine with 32 virtual cores, 128 GB of RAM, and 256 GB of local (SSD) storage. The machine runs Ubuntu version 18.04, Sklearn version 0.21.2, and ml.net version 1.2. Both ML libraries were run with multithreaded. ${\sf MASQ}$MASQ was evaluated on MySQL version 5.7.29 and SQL Server 2017 version 14.0.3223. We run all experiments 5 times, and report the average. For ${\sf MASQ}$MASQ we average the query time as reported on the database catalog; for Sklearn and ml.net we time the execution within the running process. The experiments do not include the time required to convert an ML pipeline into a SQL implementation. This operation is performed offline once, and in all experiments the time taken for conversion is insignificant. Due to space limits, for some experiments, we only report MySQL numbers. Interested readers can refer to the technical report [65] for SQL Server results, as well as additional experiments.

### A. Accuracy

The first step for evaluating whether DBMSs can be used as prediction serving systems is to check that the prediction outcomes match the original ones generated by the ML framework. Rounding errors introduced by the different floating point operation implementations can in fact lead to inconsistent results [66]. In Table V we report the errors between the outcomes generated by the baseline frameworks (Sklearn and ml.net) and ${\sf MASQ}$MASQ. We compute errors as the mean of the absolute differences between the returned values (posterior probabilities of the labeled class) for regression (classification) tasks. As we can see from the table, using SQL queries for inference introduces negligible errors (e.g., between $1e-05$1e-05 and $1e-06$1e-06 in the general case; $1.49e-02$1.49e-02 in the worst case). The worst case is due to the *Compiler* which uses ml.net tree-aggregation logic, while XGBoost uses a specific aggregation function for Tweedie.

**TABLE V:** Error (Mean of the Absolute Differences) on the Predictions Generated by ${\sf MASQ}$MASQ Versus ml.net and Sklearn

Pipeline	${\sf MASQ}$MASQ vs ml.net	${\sf MASQ}$MASQ vs Sklearn	Pipeline	${\sf MASQ}$MASQ vs ml.net	${\sf MASQ}$MASQ vs Sklearn
P1	$5.99e-08$ 5.99e-08	$1.97e-06$ 1.97e-06	P2	$2.47e-06$ 2.47e-06	$2.44e-06$ 2.44e-06
P3	$3.74e-05$ 3.74e-05	$2.49e-06$ 2.49e-06	P4	$2.38e-05$ 2.38e-05	$2.50e-06$ 2.50e-06
P5	$1.65e-05$ 1.65e-05	$2.52e-06$ 2.52e-06	P6	$2.69e-05$ 2.69e-05	$1.49e-02$ 1.49e-02
P7	$1.03e-05$ 1.03e-05	$2.50e-06$ 2.50e-06	P8	$5.17e-06$ 5.17e-06	$3.81e-06$ 3.81e-06
P9	$2.07e-06$ 2.07e-06	$2.13e-06$ 2.13e-06	P10	$7.46e-06$ 7.46e-06	$1.83e-06$ 1.83e-06

### B. Throughput

**Fig. 9.**
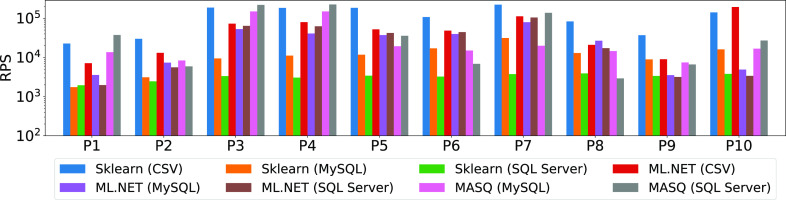
Throughput for Sklearn, ml.net (on CSV, MySQL and SQL Server) and ${\sf MASQ}$MASQ (on MySQL and SQL Server).

The goal of this experiment is to compare the performance of each framework and on each pipeline in serving predictions over the full datasets. For Sklearn and ml.net we also test the performance when the data resides both over flat CSV files and in the databases. In the latter case, data has to be moved out of the database into CSV format before the predictive pipeline can be executed. This latter case simulates what happens in practice ML deployments where data must be moved out of the database in order to be fed to the model. Since the datasets used for the pipelines have different sizes, we plot the average throughput in terms of *rows evaluated per second* (RPS). Fig. 9 shows the results.

*Discussion.* There are several insights from this experiment: (1) there is no system constantly outperforming the others (Sklearn on CSV is better on 6 over 10, ml.net on 1, and ${\sf MASQ}$MASQ on 3); (2) as expected, the throughput for the ML frameworks when the data needs to be moved out of the database decreases, although it decreases considerably (around 10×) for Sklearn, less for ml.net—we think that this is due to the quality of connectors; (3) in general there is no clear winner between MySQL and SQL Server connectors for ml.net, whereas for Sklearn, the SQL Server connector performs worse than the MySQL one; (4) ${\sf MASQ}$MASQ throughput is better than the database version of the ML frameworks for almost all the pipelines with linear models (P1, P3, P4), while it is slightly lower for a couple of tree ensemble models (P6, P9), and comparable to the other pipelines (P2, P5, P7, P8, P10); (5) MySQL and SQL Server implement different optimization strategies whereby the same query generated by ${\sf MASQ}$MASQ can result in a different performance. The 4th point is somehow surprising and invalidates the common knowledge that databases are not performant over linear algebra. Conversely, tree-model performance varies based on the implementation and dataset. We will further explore this behavior in the following sections.

### C. Scalability

**Fig. 10.**
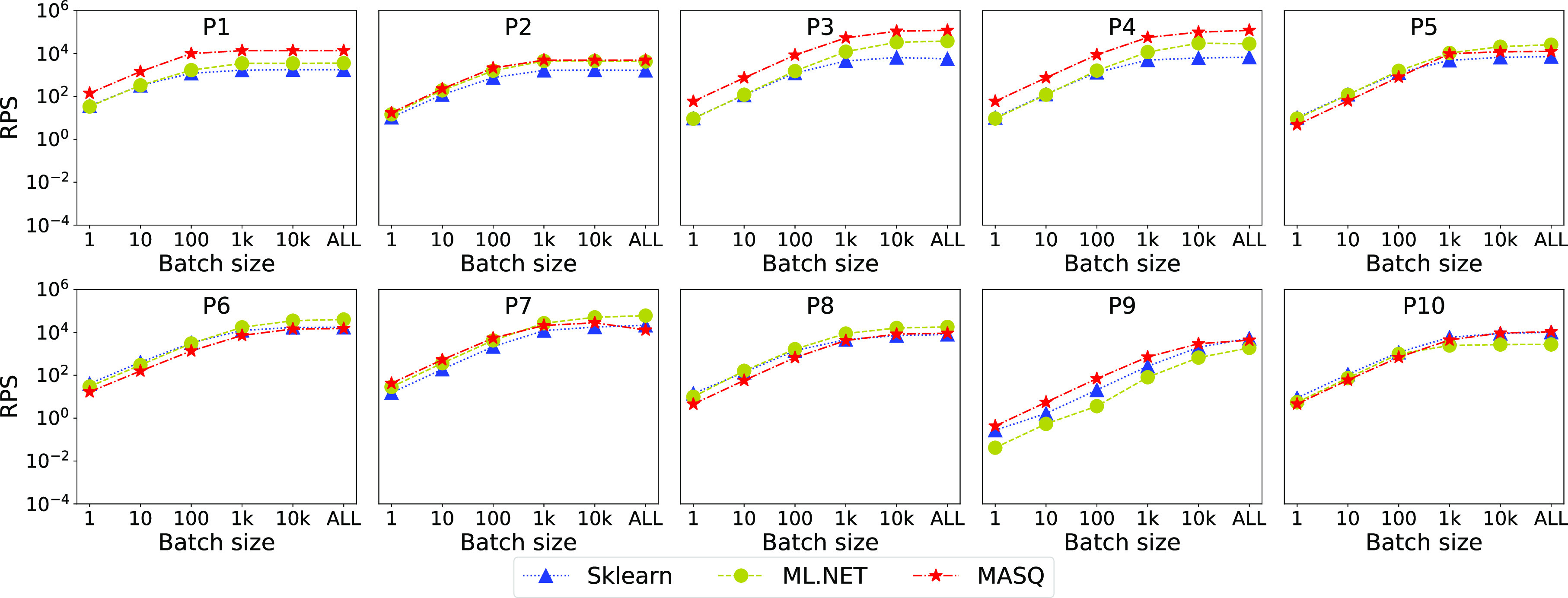
Scalability of the different frameworks, over MySQL, as we change the batch size.

In this section, we study how the throughput changes as we scale the data processed by each system. We implement this scenario by splitting each dataset into batches of various sizes, and plotting the overall throughput. We test batches of 1 (i.e., online predictions), 10, 100, 1 K, and 10 K rows, plus the full dataset in one batch. We use the full dataset in cases where the batch size is greater than the total length. Fig. 10 shows the results for MySQL. For Sklearn and ml.net we run the versions where the data resides in the database.

*Discussion.* We can notice similar trends in all the pipelines: as the batch size increases, the throughput increases as well, up to a saturation point (either we saturate over the dataset size or the resources). Regarding ${\sf MASQ}$MASQ: for pipelines P1, P3, P4, and P7, with linear models, ${\sf MASQ}$MASQ shows the best performance in most of the settings. Conversely, for tree-based models, we can see that in the majority of the settings (P2, P5, P9, P10) ${\sf MASQ}$MASQ performance is either the best or in between Sklearn and ml.net. For the remaining pipelines (P6, P8) ${\sf MASQ}$MASQ trend is generally worse than the baseline frameworks, although in aggregate not by much. This is because these pipelines have tree ensemble models and either simple, or absent featurization. In this case, we cannot use optimizations as we are doing for other tree-based pipelines.

### D. Latency

**Fig. 11.**
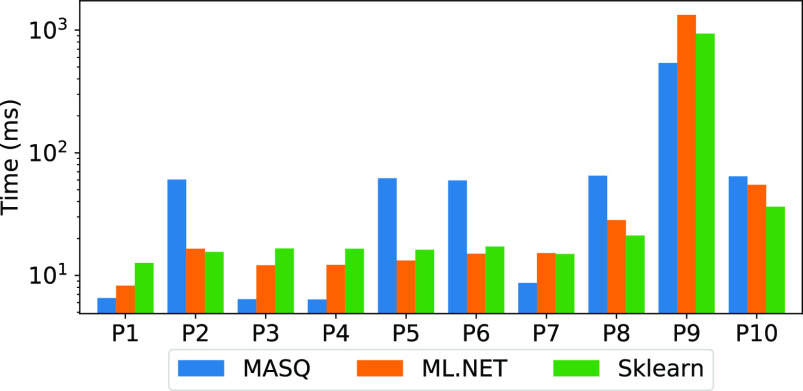
Latency numbers over a single record (MySQL).

In this section, we focus on the latency performance for executing online (single record) predictions. Fig. 11 shows the results computed over MySQL, where for ml.net and Sklearn we also consider the time to pull the records out of the database.

*Discussion.* The latency numbers confirm that ${\sf MASQ}$MASQ performs better (up to 3×) than the baseline frameworks for linear models (P1, P3, P4, P7) while tree-based models (P2, P5, P6, P8, P9, P10) can be up to around 2× slower (P2, P5, P6). Even for the same dataset, we can notice the latency of tree ensemble models is worse than the linear ones (i.e., P4, P5). Next, we will study more in detail the trade-offs between linear and tree ensemble models by breaking down the performance for each single pipeline component.

### E. Performance Breakdown

In this Section, we drill down into the performance of a few selected queries over the largest datasets. We first evaluate how each pipeline operator contributed to the final runtime for queries P7, P8, P9 and P10 (Section V-E1). Successively, we further look deeper into how time is spent between data loading, data writing, and computing for all the above pipelines (Section V-E2).

#### 1) Operator Breakdown

**Fig. 12.**
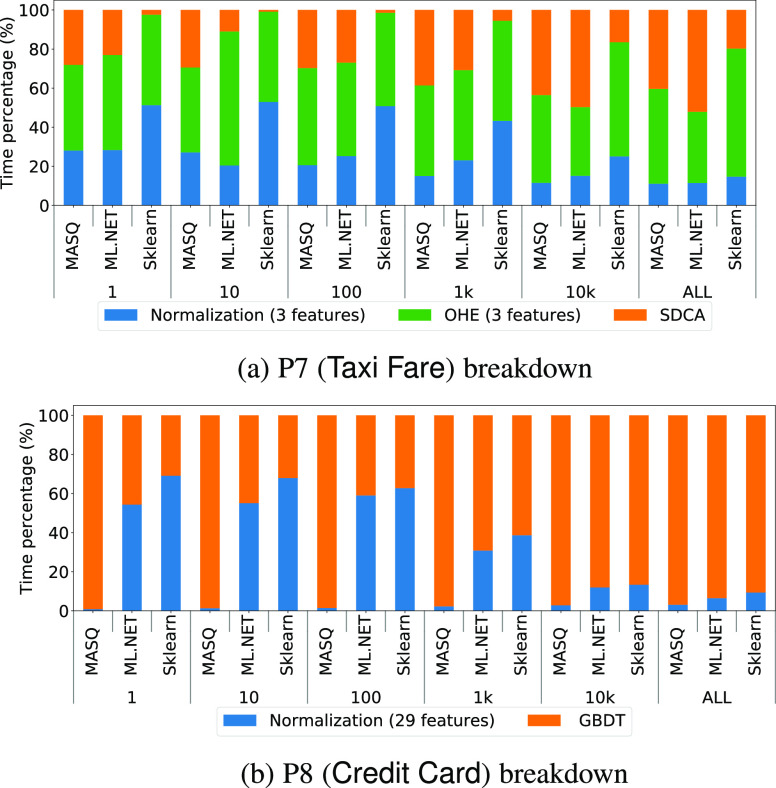
Operator breakdown for P7 and P8.

**Fig. 13.**
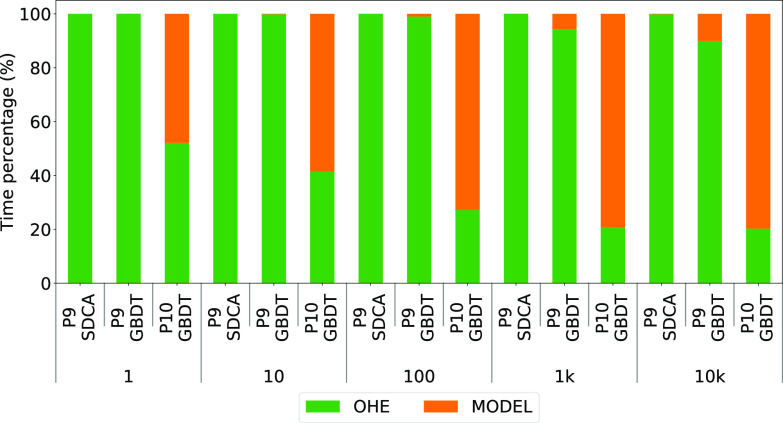
Operator breakdown for P9 (${\sf Criteo}$Criteo) and P10 (${\sf Flight Delay}$FlightDelay). For P9 we also compare GBDT vs SDCA.

We plot, by batch size (where a batch of 1 is online), the runtime for each operator as a percentage of the total runtime. For ${\sf MASQ}$MASQ we report the numbers over MySQL (similar results hold for SQL Server), and we compare it against Sklearn and ml.net over CSV for P7 and P8 in Fig. 12. In Fig. 13 we instead report the results for ${\sf MASQ}$MASQ for P9 and P10, where for P9 we show two variants: one with a tree model (GBDT, as described in Table IV) and one with a linear model (SDCA). Recall that ${\sf Criteo}$Criteo is the largest dataset with 2.5 M features (after OHE). We run two variants so that we can study, in the worst-case scenario, the tradeoffs between linear and tree ensemble models for ${\sf MASQ}$MASQ.

*Discussion.* Starting with P7, we notice that: (1) data featurizers take the majority of the time; and (2) as the batch size increases, the time spent on normalization decreases. This second point is even more marked on P8 where for Sklearn and ml.net normalization surprisingly takes more than 50% for batches of 1, while it takes less than 10% when we score the entire dataset at once. We think that this behavior is due to the benefits of vectorization which increases with the batch size. In P8, for ${\sf MASQ}$MASQ we see that the majority of the time ($>$>90%) is spent on the evaluation of the GBDT model.

If now we move our attention to the evaluation of P9 and P10 in Fig. 13, we see that: (1) the time required to complete the OHE operator is proportional, as expected, to the number of features generated rather than the number of rows processed (i.e., the percentage of time spent on OHE is greater in P9 than in P10: the first generated 2.5 M features over 4 M rows, the second 700 over 21 M rows); (2) as the batch size increase, the time spent on executing the GBDT model increases, up to reach 80% in P10 for a batch size of 10 K. The experiment performed on P9 with SDCA, instead, confirms that the time required to execute the linear model is irrelevant wrt the time for executing the featurizer or the GBDT.

#### 2) Latency Breakdown

In this section, we look at the latency (single record) performance for the pipelines used in the previous section. We compute the breakdown by dividing the latency into three components: *data loading*, *computation*, and *data writing*. For this experiment, we have enabled the profiling of all events / statements generated by the queries using SET @@ profiling = 1 and SET @@ profiling history size = 100, and we classified each event using the above components. We report the time performance measured on ${\sf MASQ}$MASQ running on MySQL, and compare it against Sklearn and ml.net run both over CSV files, and when records are loaded from MySQL.

**Fig. 14.**
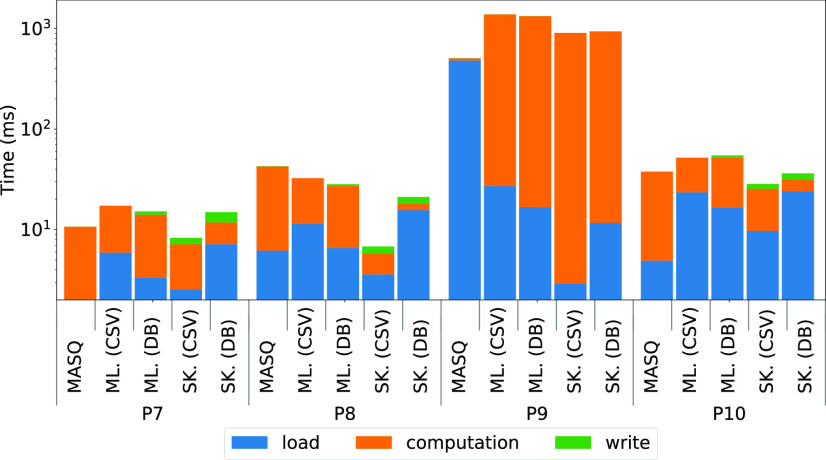
Latency breakdown for ${\sf MASQ}$MASQ, ml.net (ML.) and Sklearn (SK.), for pipelines P7, P8, P9 and P10. The time spent is divided into three buckets: load, computation, and write.

*Discussion.* The breakdown in Fig. 14 shows that the computation time is dominant in P7 for ${\sf MASQ}$MASQ, while load takes the majority of time for P9. We think that this is because P7 contains a linear model whereby the majority of the time is spent in multiplications, while for P9 the one-hot encoder generates 2.5 million features out of 26 columns, thus creating substantial data access in our implementation.

For ml.net and Sklearn the computation time is almost always dominant. Interestingly, the difference between data loading for the CSV and the DB is minimal for ml.net while it is quite large for Sklearn. Again, we think that this is due to the quality of the database connectors.

### F. Optimizations

In this section, we explore database-specific optimizations such as adding indexes (Section V-F1) and “logical optimizations” at the operator level such as operator fusion (Section V-F2).

#### 1) Using Indexes

**Fig. 15.**
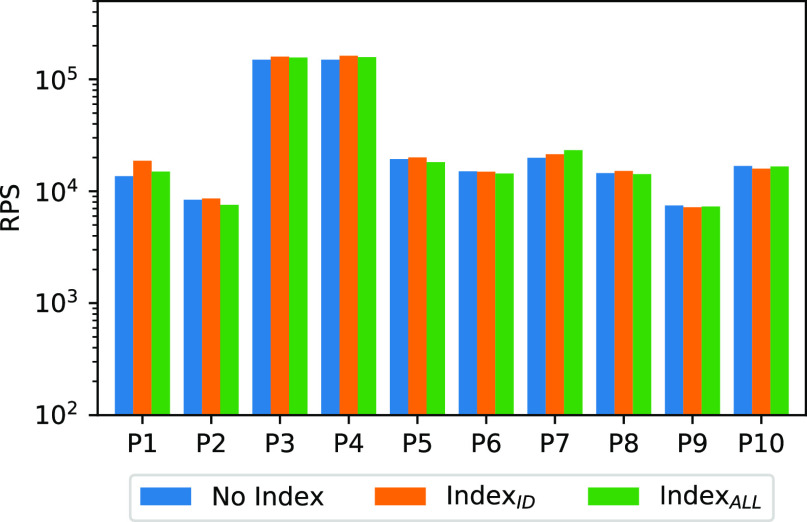
Performance comparison with indexing (MySQL).

In this experiment, we evaluate whether the performance over the DBMSs can be improved by applying indexes. We evaluate three settings: in the first setting, referred to as *No Index*, we add a clustered index on the primary key. In the second setting, *Index$_{ID}$ID*, a non-clustered index is added to the column identifier ($ID$ID). Finally, in the setting *Index$_{ALL}$ALL* we add a non-clustered index for each column. We add indexes both to the input dataset, and to temporary tables when used (e.g., in P7, P9 and P10). Fig. 15 contains the results of this experiment for MySQL. SQL Server results are similar.

*Discussion.* The results show that there is no benefit from indexing. This indeed is unexpected behavior. Our intuition is that the secondary indexes should help, for example, on tree models for retrieving records more efficiently. This is because, in each decision node, we only fetch records with specific conditions. However, as the experiment shows, this is not the case. We think that this is because conditions are expressed in case statements that cannot be pushed into index lookups. Additionally, we do not see any improvement for the pipelines where indexes are built also on temporary tables (P7, P9 and P10). Note that in this latter case, the cost of building the index is counted into the running time of the final queries. We also explored a column-store layout in SQL Server. What we found is that, similarly to indexing, this technique does not introduce any significant improvement and sometimes even degrades performance. With small batches (i.e. 1 to 10 k) we measured performance degradations of up to 3×. This is due to the overhead of reconstructing the per-row format of records. With large batches (i.e. greater than 100 k) instead we found an increase in performance only in P8 and with a tree ensemble depth greater than 6. This is motivated by the fact that deep trees require repeated access to the features and this pattern is able to better exploit the columnar format.

#### 2) Operator Fusion

**Fig. 16.**
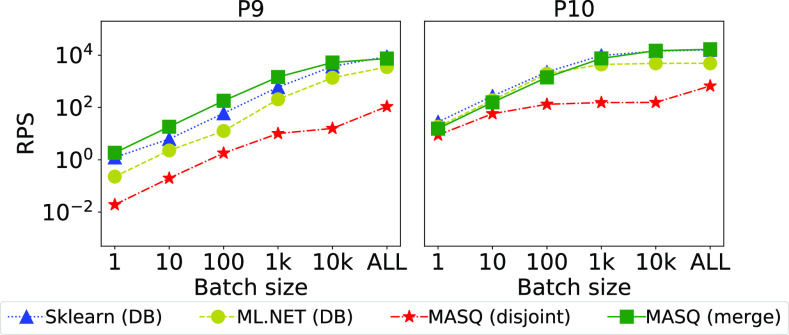
Operator fusion (OHE + GBDT) for P9 and P10.

We evaluate an optimization for pipelines P9 and P10, where the queries implementing the tree ensemble models are fused with the OHE. Specifically, the case statements evaluating the tree conditions on the columns targeted by the OHE are rewritten to compute both the featurization and the prediction in the same statement. Note that this optimization is currently not supported by database optimizers, and therefore we had to manually implement it directly in ${\sf MASQ}$MASQ. Fig. 16 shows the RPS for the optimized implementation on MySQL compared to the baseline where no optimization is used.

*Discussion.* The results show that when operator fusion is not used ${\sf MASQ}$MASQ performance decreases substantially for P9 and P10. With operator fusion, ${\sf MASQ}$MASQ does more computations per single row (i.e., for each row we compute the encoding multiple times, one for every time the row is used by a tree), but since the number of features is large and not all of them used, the total number of encodings is less. We tried a similar optimization for P8 where we fuse normalization with GBDT. This last experiment introduced a 4× slowdown. This is because all features are used by the GBDT model. This result suggests that a cost-based optimizer is likely required for selecting the best compilation strategy when optimizations are enabled. Regarding latency, operator fusion improves P9 by 5×, and P10 by 2×.

### G. Study of Operators Implementation

In this section, we study a few possible variants of the operator implementations discussed in Section IV-A4 (Section V-G1 for tree ensembles, and Section V-G2 for linear models) as well as how model characteristics affect the query performance (Section V-G3).

#### 1) Tree Ensembles Implementation

**Fig. 17.**
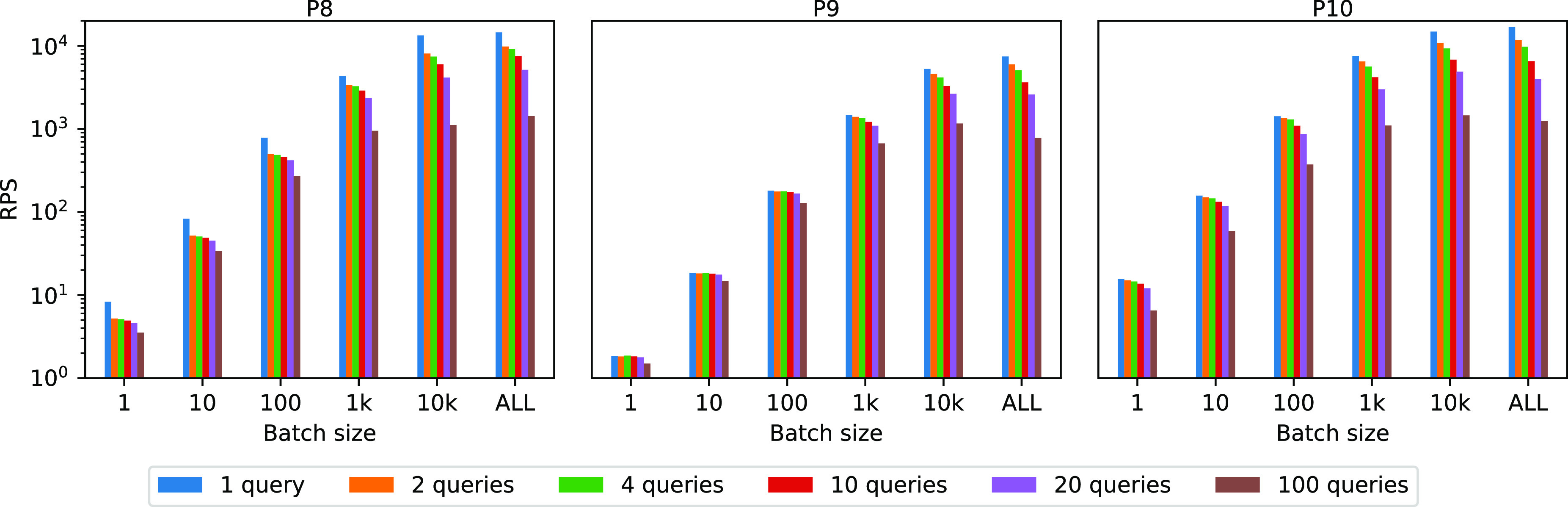
Comparison of different tree implementation methods (MySQL).

Pipelines P2, P5, P6, P8, P9, and P10 make use of tree ensemble algorithms whereby a certain number of trees (100 in our experiments) are executed, and their predictions combined. In this experiment, we test two different implementations for this operation. In the first implementation, the queries representing each tree are a subquery of an outer query computing the final score over the partial results (this is the approach described in Example 7). The results obtained with this implementation are represented in Fig. 17 as “1 query”. In the second implementation, we batch different sets of trees (1, 5, 10, 25, and 50) in multiple queries (respectively 100, 20, 10, 4, 2) and store the partial predictions into an intermediate table. A final query then computes the output by aggregating the results from the temporary table. In this experiment, we use pipelines P8, P9, and P10, and we tested over different batch sizes. In Fig. 17 we plot the results for MySQL.

*Discussion.* The experiment shows that the approach with a single query outperforms the others in pretty much any setting. This is because the database is able to optimize the execution end-to-end using a single query, while the more queries we use, the less they can be optimized.

#### 2) OHE Followed by Linear Models

**Fig. 18.**
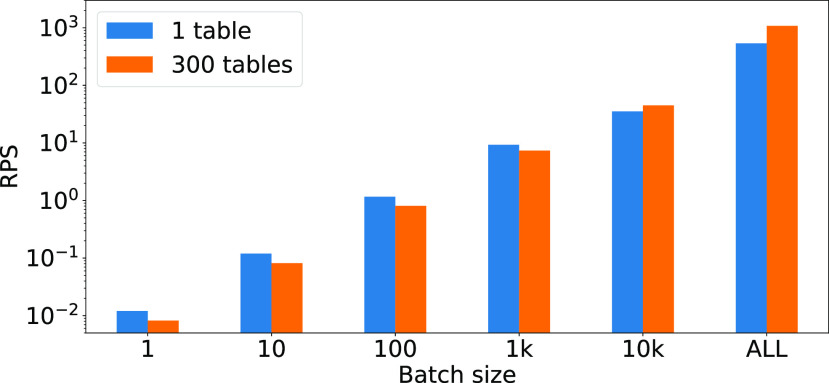
Comparison of single intermediate data and multi-way join strategy for OHE + linear models.

When an OHE featurizer is followed by a linear model, a temporary table is built storing the results of the featurization (the ${\sf OHETable}$OHETable in Fig. 7), and its content is joined with the model parameters table (see Section IV-A4 for details). In this experiment, we evaluate a possible alternative plan for implementing the operation as a multi-way join. We perform a test on MySQL against pipeline P9 with SDCA, where the ${\sf OHETable}$OHETable is partitioned into 300 tables.^fn7^^7^This is the minimum number of tables required in order to meet MySQL limits on ${\sf case}$case statements. Fig. 18 plots the results of the experiment over different batch sizes.

*Discussion.* As we can see the join implementation performs better over large batch sizes, whereas when the data to process is smaller, the single intermediate table implementation performs better. This is likely because, for small batch sizes, fewer inserts to the intermediate table are executed concurrently.

#### 3) Tree Ensembles With a Variable Number of Leaves

**Fig. 19.**
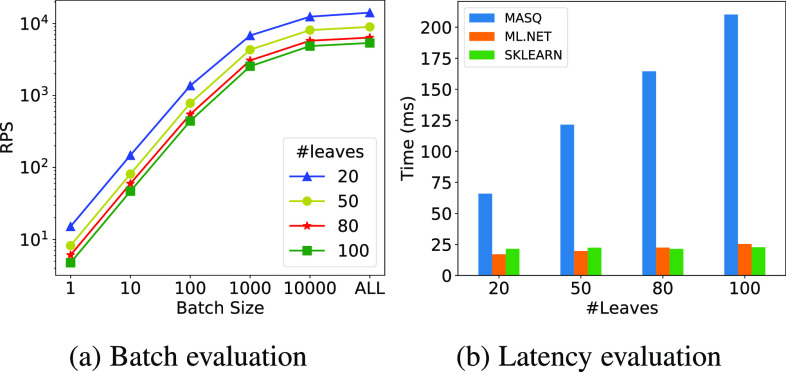
Comparison of tree ensembles performance with variable number of leaves on P8.

In this experiment, we study how the performance of our tree ensemble implementation varies as we increase the number of leaves (i.e., the height) of the trees. Fig. 19(a) and (b) report the performance on MySQL of different P8 tree ensembles implementations obtained by varying the number of leaves.

*Discussion.* Fig. 19(a) shows how performance varies, per batch size, as we increase the number of leaves. As we can see, the difference in performance is stable across the different batch sizes, and it is because evaluating taller trees (with more leaves) requires the evaluation of more conditions. If we look specifically at the batch size of one, from Fig. 19(b) we can conclude that P8 latency is from 3× to 6× slower on ${\sf MASQ}$MASQ compared to the baseline systems. Interestingly, Sklearn and ml.net performance slightly increases with the increase of the number of leaves, while ${\sf MASQ}$MASQ gets up 2× slower. This is likely due to the overhead of unrolling tree ensemble evaluation as a sequence of case statements.

### H. Negative Results

In this section, we consider two scenarios that are common in ML pipelines but we found to be hard to support in databases, with reasonable performance: featurization of textual data (Section V-H1), and neural network models (Section V-H2).

#### 1) Managing Textual Data

**Fig. 20.**
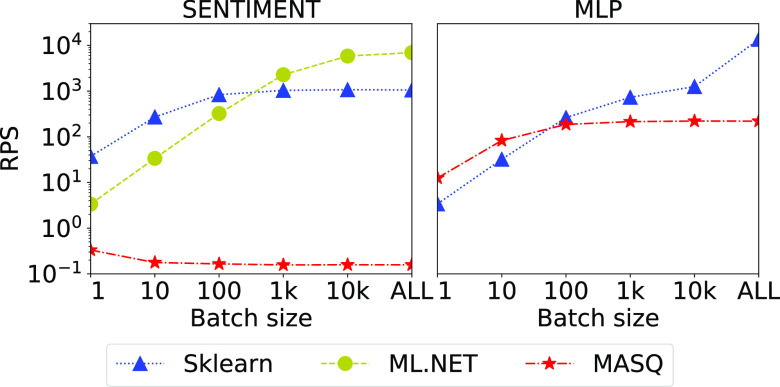
Left hand-side: Sentiment Analysis over textual features. Right hand-side: an MLP model applied on ${\sf Credit Card}$CreditCard.

To study whether ${\sf MASQ}$MASQ can support textual data, we create a pipeline over the ${\sf Sentiment}$Sentiment dataset [67]. This dataset contains 40 k records, with 7 numerical and 1 textual feature each. The ML pipeline is composed of a data featurizer (*FeaturizeText* in ml.net, *TfidFeaturizer* in Sklearn) over the textual column, and a logistic regression model. After the application of the text featurizer, the number of features becomes around 210 K. We implemented the text featurizer in SQL using temporary tables and case statements, while the logistic regression is implemented as a simple select statement. The left-hand-side plot in Fig. 20 shows the results against MySQL. Sklearn and ml.net are run over the data stored in the database.

*Discussion.* The experiment shows that ${\sf MASQ}$MASQ performance is several orders of magnitude off compared to the baseline frameworks. This is due to: (1) the large number of features generated; (2) the implementation of the text featurizer which mixes case statements and temporary table transformations; and (3) the heavy use of the string intrinsics provided by the database. We believe that text featurizers are better supported in databases with UDFs.

#### 2) What About Neural Networks?

For this experiment, we created a SQL implementation of a Multilayer Perceptron (MLP) through select statements. We test the implementation using a simple model composed of 3 hidden layers, each one with 5 nodes. We used the ${\sf Credit Card}$CreditCard dataset for the experiment, and we compare the results with Sklearn (note that ml.net currently does not provide native support for MLP models). The results for MySQL are plotted on the right-hand side of Fig. 20.

*Discussion.* As we can see from the results, ${\sf MASQ}$MASQ performance is comparable to Sklearn only for small batch sizes, whereas for larger batch sizes Sklearn is able to better use the hardware than MySQL. This MLP model requires three matrix multiplications, and Sklearn uses BLAS libraries to efficiently compute them. Note that these results are over a very small MLP with only 3 layers and 5 neurons per layer. We also experimented with larger MLPs with a few hundred neurons, and the results are, as expected, worse by several orders of magnitude.

## VI. Lesson Learned and Conclusions

From this experimental evaluation, we learned several interesting insights. For example, linear models are not a bottleneck, while featurizers and tree-based models can be. Adding indexing is not helpful, while operator fusion sometimes is. Furthermore, we had to come up with specific implementations and optimizations to address database limits, and these scenarios can be quite common in practice. ${\sf MASQ}$MASQ currently supports more than a dozen of featurizers and models (Section IV-A3), and we are actively working on adding support for additional operators (e.g., feature selection operators, imputers, K-means, missing linear and tree models). Unfortunately, while we believe that any ML operator can be translated into SQL, we are aware that not all operators will have good performance, as we saw for text featurization, and neural networks. We think that, to properly support these operators, a UDF-based approach is probably required. Additionally, since many operators (e.g., tree methods and one-hot encoding) use case statements, having better support for deep case expressions will probably help with the performance.

Finally, we discovered several interesting compromises between optimizations and compilation strategies. Examples are, when to use operator fusion (Section V-F2), or when to change the operator implementation (Section V-G). This suggests that a cost-based optimizer is likely required to achieve the best performance. This is even more true when hardware accelerators are also available [53]. We recently started the exploration of this exciting space [44], [54], [68].
